# Impact of central sensitization on pain, disability and psychological distress in patients with knee osteoarthritis and chronic low back pain

**DOI:** 10.1186/s12891-023-07019-z

**Published:** 2023-11-10

**Authors:** Doha Dahmani, Fatima Zahrae Taik, Imane Berrichi, Maryam Fourtassi, Fatima Ezzahra Abourazzak

**Affiliations:** 1 Rheumatology Department, Faculty of Medicine and Pharmacy, Mohammed VI University Hospital, Abdelmalek Essaadi University, Tangier, Morocco; 2 Laboratory of Life and Health Sciences, Faculty of Medicine and Pharmacy of Tangier, Abdelmalek Essaadi University, Tangier, Morocco

**Keywords:** Central sensitization, Central sensitization inventory, Knee osteoarthritis, Chronic low back pain

## Abstract

**Background:**

Central sensitization (CS) is becoming increasingly recognized as a significant factor in many chronic pain conditions, including knee osteoarthritis (KOA) and chronic low back pain (CLBP). Yet it presently remains unclear how strong is the involvement of CS in KOA and CLBP and which factors are involved in CS in these two chronic disabling diseases.

**Methods:**

This is a cross-sectional study in which included a total of 178 patients with KOA and 118 patients with CLBP. Inclusion criteria for eligible participants for the KOA group were a confirmed diagnosis of KOA according to the American College of Rheumatology criteria, and for the CLBP group a chronic low back pain for more than 3 months. Subjects were excluded if they presented with a diagnosed psychiatric disorder or if they lacked the capacity to provide informed consent, understand study questionnaires or perform physical performance tests. In each group, were assessed; CS-related symptoms using the Central Sentization Inventory (CSI); demographic and clinical characteristics such as disease duration, pain intensity on a visual analog scale, self-reported function using the Lequesne index for KOA patients and the Oswestry Disability index for CLBP patients, and physical performance with the 6 minutes’ walk test; as well as psychosocial risk factors using the Patient Health Questionnaire for depression (PHQ-9), the Generalized Anxiety Disorder (GAD-7) and the Pain Catastrophizing Scale (PCS).

**Results:**

CSI scores significantly correlated with pain intensity and disability in KOA and CLBP patients, and were highly correlated with self-reported symptoms of depression, anxiety and pain catastrophizing. Depression significantly predicted the CSI score in both groups.

**Conclusion:**

These findings provide further evidence for the impact of CS on pain, function and physical performance in KOA and CLBP patients. Psychosocial symptoms such as pain catastrophizing, anxiety and depression should also be considered as they are also associated with CS.

## Background

Knee osteoarthritis (KOA) and chronic low back pain (CLBP) represent a major cause of pain and disability worldwide, with high costs for the patient, his family, and the society [1, 2]. However, their treatment remains challenging as the underlying pain mechanisms are not fully understood [3]. As there is often a disparity between the chronic pain intensity and the severity of the tissue damage, health care professionals tend to underestimate the pain intensity as compared to what is actually reported by the patients [4]. In KOA, the pain associated with joint disease is different from one patient to another, and shows only a weak association with radiologic imaging features [5, 6]. A complete absence of identifiable pathoanatomical alterations is common in patients with CLBP [7, 8]. Consequently, the degree of spine damage as measured by radiographs, MRI or CT-scans is not correlated with the presence or severity of pain [9].

One reason for this disparity and the unproportionally high pain experience is neuroplastic changes that occur in the peripheral and central nervous system and result in pain sensitization enhancing the nociceptive drive from a damaged structure and hence causing more pain than can be accounted for by the damage [10–14]. In summary, the pathophysiological mechanisms underlying central sensitization (CS) are numerous, including an upregulation of nociception from enhanced synaptic transmission secondary to loss of spinal cord inhibitory inter-neurons, a facilitation of ascending pain mechanisms, an alteration of the descending inhibitory pathways, a facilitation of cognitive-affective mechanisms, and an altered cortical processing of nociceptive inputs [11, 15–19]. These processes result in a heightened and long-lasting response to painful stimuli, and may also lead to non-noxious stimuli being interpreted as painful [11, 15–19].

If direct electrophysiological recordings from central neurons have proved the existence of CS in animal studies, these methods cannot be performed in humans [14]. However, the conclusions drawn from animal studies were used to explain similar pain phenomena in human beings, leading to the introduction of the term ‘human assumed central sensitization’ (HACS) [20]. Several clinical signs and symptoms may indicate HACS, and various methods have been considered for its quantification in human patients. These include questionnaires [21], quantitative sensory testing (QST) [22] including mechanical pressure and injection of hypertonic saline [23, 24], functional Magnetic Response Imaging (fMRI) [25], and brain-derived neurotrophic factor analysis (BDNF) [26, 27].

The Central Sensitization Inventory (CSI) was developed as a screening tool to assess CS-related symptoms in a number of chronic pain conditions [21], including KOA and CLBP. It has been translated into numerous languages [28–33] and widely adopted in scientific research and clinical practice. Yet it presently remains unclear how strong is the involvement of CS in KOA and CLBP and which factors are affecting the central pain processing in these two chronic disabling diseases. A better understanding and identification of these factors and their impact on the patient’s condition is therefore important for clinicians in order to work toward a tailored treatment strategy.

The aim of the present study was twofold: (1) to analyze the associations between the CSI score, and pain-related symptoms, self-reported function, physical performance and psychosocial factors in KOA and CLBP patients, (2) to explore potential factors that contribute to CS in these two patients’ groups.

## Methods

### Study design and setting

This is a cross-sectional study carried out in the department of rheumatology of Tangier’s university hospital in Morocco, between February 2022 and September 2022. Data was prospectively collected from the outpatient department. The study was approved by the ethics committee of Tangier’s university hospital (n 01/2022), and all procedures performed on this study were in accordance with the ethical standards of the 1964 Helsinki declaration. Written informed consent was obtained from all subjects prior to the study.

### Participants

Inclusion criteria for eligible participants for the KOA group were a confirmed diagnosis of KOA according to the American College of Rheumatology criteria, and for the CLBP group were a chronic low back pain for more than 3 months. Subjects were excluded if they presented with a diagnosed psychiatric disorder or if they lacked the capacity to provide informed consent, understand study questionnaires or perform physical performance tests.

### Data measurement

#### Sociodemographic data

Personal variables as age, sex, body mass index (BMI) and education level were collected.

#### Disease characteristics

Duration of disease was measured as the number of years of disease progression. Radiographic evidence of KOA was graded according to the Kellgren-Lawrence scale. The number of co-morbidities was defined. Pain intensity was rated on a visual analog scale (VAS). The Arabic version of the Lequesne index was used to assess function in KOA group [34], while the Arabic version of the Oswestry Disability Index (ODI) [35] was used to assess function in CLBP patients. The six-minute walk test (6MWT) [36] evaluated physical performance in both groups.

#### Central sensitization

Pain sensitization was assessed using the Arabic version of the Central Sensitization Inventory (CSI). The CSI consists of two parts: A and B [21]. Part A contains 25 items presenting the pain related psychosocial, cognitive and functional items. Each item is rated on a 5-point Likert-type scale (0 = never and 4 = always). Part B (which is not scored) is designed to determine whether one or more specific disorders, including seven separate Central Sensitivity Syndromes (CSS), had been previously diagnosed (restless leg syndrome, chronic fatigue syndrome, fibromyalgia, temporomandibular joint disorder, migraine or tension headaches, irritable bowel syndrome, multiple chemical sensitivities, neck injuries (including whiplash), anxiety or panic attacks, and depression). Part B was not used in the present study. The total CSI score was obtained by summing the scores of the 25 individual items of part A. Higher total CSI scores reflect higher CS self-reported symptomatology (0–29 : sub-clinical, 30–39 : mild, 40–49 : moderate, 50–59 : severe, and 60–100 : extreme) [37]. A 40-points score out of 100 was described as the cut off value, indicative for CS [38]. The Arabic version of the CSI has shown good psychometric properties for test-retest reliability (ICC: 0.699) and internal consistency (Cronbach’s alpha: 0.823).

#### Psychosocial self-reported measures

Patients completed a number of validated self-reported questionnaires for measuring depression, anxiety, and pain catastrophizing using the Arabic version of the Patient Health Questionnaire-9 (PHQ-9) [39], the Generalized Anxiety Disorder (GAD) score [40] and the Pain Catastrophizing Scale (PCS) respectively. The Arabic version of the PCS has shown good psychometric properties for test-retest reliability (ICC: 0.583) and internal consistency (Cronbach’s alpha: 0.736).

#### Physical activity

Physical activity was assessed using the Arabic version of the International Physical Activity Questionnaire (IPAQ) [41] and by identifying the daily sedentary time (in minutes).

### Sample size calculation

Using an expected correlation coefficient of 0.3 between CSI and the different predictors [42–46], with an 80% power (β = 0.2) and a 5% significance level two-sided test (*α* = 0.05), a minimum sample size of 85 patients in each group was required for this study [47].

### Statistical methods

Descriptive characteristics are expressed using percentages (%) for categorical variables, means ± standard deviation (SD) for normally distributed continuous variables, and median first and third quartile (Q1, Q3) otherwise. To analyze the correlation of pain intensity, self-reported function, physical performance and psychosocial factors with the CSI score, we performed univariate correlation analysis using Pearson correlation coefficients in each group. Simple linear regression was used to identify variables that significantly predicted the CSI. Relationships between candidate variables and the CSI score were tested using multiple linear regression. The results are expressed in terms of unstandardized beta coefficients with 95% confidence intervals (ß, 95% CI) and p values. All analyses were conducted using SPSS V.21. The level of significance was set at *p* < 0.05.

## Results

In this study, 178 patients with KOA and 118 patients with CLBP were included.

### Demographic and clinical characteristics of KOA and CLBP patients

The participants’ characteristics are summarized in Table 1.

**Table 1:** Demographic and clinical characteristics of participants

	KOA (n = 178)	CLBP (n = 118)
Age (years)	60 (52, 64)	52.50 (42, 66)
Sex		
F M	157 (88.2%) 21 (11.8%)	102 (86.4%) 16 (13.6%)
Number of co-morbidities	2 (1.75, 3.25)	3 (1, 3)
Education level		
Low Elementary High school University	127 (71.3%) 24 (13.5%) 18 (10.1%) 9 (5.1%)	73 (61.9%) 21 (17.8%) 12 (10 0.2%) 12 (10.2%)
BMI kg/m^2^	30.45 (26.98, 34.90)	27.55 (24.78, 31.20)
Duration of disease (years)	3 (1, 8.50)	5 (2, 10)
Kellgren-Lawrence score	2 (2, 3)	
Pain intensity	5 (3, 6)	6 (5, 7)
Self-reported function		
Lequesne index ODI: Mild disability (%) Moderate disability (%) Severe disability (%)	9.8 ± 3.88 - - -	- 28.6 48.6 22.9
6MWT	377 (297.20, 475.13)	386.36 (297.00, 493.45)
Daily sedentary time (minutes/day)	120 (60, 180)	120 (60, 240)
IPAQ	2051.25 (488.38, 4265.25)	1404 (297.00, 3826.50)
PHQ-9	8 (5, 12)	8 (4.75, 13)
GAD-7	8.50 (4, 14)	9 (5, 14)
PCS	23 (12, 38)	28 (13, 44)
CSI-A	38.95 ± 16.81	42.16 ± 17.67
CSI-A ≥ 40	84 (47.2%)	63 (46.6%)

KOA: Knee Osteoarthritis; CLBP: Chronic Low Back Pain; BMI: Body Mass Index; ODI: Oswestry Disability Index, 6MWT: Six-Minute Walk Test; IPAQ: International Physical Activity Questionnaire; PHQ-9: Patient Health Questionnaire-9; GAD-7: Generalized Anxiety Disorder-7; PCS: Pain Catastrophizing Scale; CSI-A: Central Sensitization Inventory part A

### Correlation of pain intensity, self-reported function, physical performance and psychosocial factors with the CSI score in KOA and CLBP

Table 2 shows the correlation coefficients with the CSI score in KOA and CLBP groups. Pain intensity correlated significantly but weakly with the CSI in KOA (r = 0.190, p = 0.012) and CLBP group (r = 0.197, p = 0.033). Self-reported function significantly correlated with the CSI score only in the KOA group (r = 0.361, p < 0.001), while the six-minute walk test for physical performance negatively correlated with the CSI score only in the CLBP group (r=-0.249, p = 0.008). Depression, anxiety and pain catastrophizing scores significantly correlated with the CSI score in both KOA (PHQ-9: r = 0.671, p < 0.001, GAD-7: r = 0.563, p < 0.001, PCS: r = 0.419, p < 0.001) and CLBP (PHQ-9: r = 0.653, p < 0.001, GAD-7: r = 0.482, p < 0.001, PCS: r = 0.411, p < 0.001) groups.

**Table 2:** Correlation of pain intensity, self-reported function, physical performance and psychosocial factors with the CSI score in KOA and CLBP patients

	KOA		CLBP	
	**r**	**p**	**r**	**p**
Pain intensity	0.190	0.012	0.197	**0.033**
Lequesne	0.361	**< 0.001**		
ODI			0.005	0.976
6MWT	-0.099	0.205	-0.198	**0.035**
PHQ-9	0.671	**< 0.001**	0.653	**< 0.001**
GAD-7	0.563	**< 0.001**	0.482	**< 0.001**
PCS	0.419	**< 0.001**	0.411	**< 0.001**

KOA: Knee Osteoarthritis; CLBP: Chronic low back pain; ODI: Oswestry Disability Index, 6MWT: Six-Minute Walk Test; PHQ-9: Patient Health Questionnaire-9; GAD-7: Generalized Anxiety Disorder-7; PCS: Pain Catastrophizing Scale

### Linear regression analysis of factors affecting the CSI score in KOA and CLBP patients

Using simple linear regression, sex, number of co-morbidities, pain-intensity, depression, anxiety and pain catastrophizing score were found to have a significant regression coefficient in both KOA and CLBP groups (Table 3). Lequesne index and age were also found to have a significant regression coefficient in the KOA and the CLBP group respectively.

For each group, only variables found to have a statistical significance in the univariate analysis were included in the multivariable linear regression, which determined that depression significantly predicted CSI score in both KOA (unstandardized ß= 1.488; CI (0.944, 2.032) p < 0.001), and CLBP patients (unstandardized ß= 1.870; CI (1.062, 2.677) p < 0.001). Number of co-morbidities was also found to significantly predict CSI score in the KOA group (unstandardized ß= 1.775; CI (0.361, 3.190) p: 0,014) (Table 3). The underlying assumptions of the multivariate regression were checked. Analytical and graphical tools confirmed the performance of our model.

**Table 3:** Regression analysis of factors affecting the CSI score in KOA and CLBP patients

	KOA				CLBP			
	**Simple linear regression**		**Multivariate regression**		**Simple linear regression**		**Multivariate regression**	
	**ß (95% CI)**	**p**	**ß (95% CI)**	**p**	**ß (95% CI)**	**p**	**ß (95% CI)**	**p**
Age	-0.202 (-0.452, 0.048)	0.113			**0.240 (0.014, 0.466)**	**0.038**	0.046 (-0.223, 0.315)	0.736
Sex	**-16.949 (-24.226, -9.637)**	**≤ 0.001**	-5.935 (-12.415, 0,546)	0.072	**-11.900 (-21.095, -2.704)**	**0.012**	-3.101 (-17.320, 11.119)	0.666
Number of comorbidities	**2.984 (1.187, 4.781)**	**0.001**	**1.775 (0.361, 3.190)**	**0.014**	**3.098 (0.802, 5.393)**	**0.009**	2.191 (-0.275, 4.657)	0.081
Duration of symptoms (Years)	0.338 (-0.064, 0.740)	0.099			0.268 (-0.141, (0.677)	0.197		
Kellgren-Lawrence score	-1.406 (-5.580, 2.769)	0.507			-	-	-	-
Pain intensity	**1.444 (0.334, 2.553)**	**0.011**	0,224 (-0.736, 1.185)	0.645	**1.676 (0.141, 3.212)**	**0.033**	0.328 (-1.293, 1.949)	0.689
Lequesne index	**1.566 (0.957, 2.175)**	**≤ 0.001**	0.542 (-0.039, 1.124)	0.067				
ODI					0.112(-7.245, 7 0.469)	0.976		
Daily sedentary time (minutes)	-0.008 (-0.032, 0.016)	0.511			-0.017 (-0.043, 009)	0.192		
IPAQ	-0.488 (-2.516, 1.539)	0.635			-0.245 (-2.679, 2.189)	0.842		
PHQ-9	**2.138 (1.782, 2.494)**	**≤ 0.001**	**1.488 (0.944, 2.032)**	**≤ 0.001**	**2.002 (1.568, 2.437)**	**≤ 0.001**	**1.725 (0.959, 2 0.491)**	**≤ 0.001**
GAD-7	**1.583 (1.237, 1.929)**	**≤ 0.001**	0.494 (-0.031, 1.018)	0.065	**1.413 (0.935, 1.801)**	**≤ 0.001**	0.015 (-0.688, 0.716)	0.967
PCS	**0.428 (0.286, 0.570)**	**≤ 0.001**	-0.035 (-0.199, 0.129)	0.670	**0.421 (0.245, 0.596)**	**≤ 0.001**	0.013 (-0.211, 0.238)	0.906

KOA: Knee osteoarthritis; CLBP: Chronic Low Back Pain; ODI: Oswestry Disability index; IPAQ: International Physical Activity Questionnaire; PHQ-9: Patient Health Questionnaire-9; GAD-7: Generalized Anxiety Disorder; PCS: Pain Catastrophizing Scale

## Discussion

Our study results suggest that central sensitization, as assessed by CSI, has a significant impact on pain, function and physical performance in KOA and CLBP patients, and that pain catastrophizing, depression and anxiety are strongly correlated with CS in these patients.

Pain intensity significantly correlated with the CSI score in both KOA and CLBP patients. This result is in line with previous studies [44, 45, 48]. CS has been suggested as one of the underlying mechanisms of pain in KOA and CLBP [11, 17, 19, 42]. In fact, repeated nociceptive stimulation from damaged tissues causes nerve endings to change, resulting in a lowered threshold, prolonged and intense response, and increased sensitivity to stimuli that would not normally elicit a response [49]. As a result, spinal cord neurons that would typically only be activated by noxious stimuli can be activated by non-noxious stimuli, leading to allodynia [50]. These neuroplastic changes indicate that pain both induces, and is partly sustained by, central sensitization.

The CSI score significantly correlated with self-reported function in KOA patients and with physical performance in CLBP patients. Several studies previously reported the same associations [13, 42, 46, 51–53]. A possible explanation for the correlation of CS with disability in KOA and CLBP might be the “fear avoidance model.” According to this theory, the development of chronic pain and disability is influenced by both neurophysiological processes related to pain sensitization and psychosocial factors such as pain catastrophizing [54, 55]. An enhanced state of sensory sensitivity along with a heightened state of alertness during a pain episode may lead to fear avoidance behavior and result in more disability [56, 57]. Previous studies reported the presence of fear avoidance in patients with KOA [56] and CLBP [58–60] and suggested its contribution to pain chronicity and disability. The long-term consequences, such as increased disability or depression, may lower the threshold for pain detection and enhance the intensity of the pain experience [56]. Some studies showed that myofascial release techniques can break this cycle by decreasing pain and trigger-point pain threshold while increasing functional ability [61].

Depression, anxiety and pain catastrophizing strongly correlated with the CSI in both KOA and CLBP patients. This result had been reported in previous studies where the relationship between CS symptoms and psychological factors was emphasized [44, 62–69]. The current finding confirms the validity of the CSI as a tool to assess CS symptomatology within a construct of general distress [21, 67]. Psychosocial and cognitive behavioral factors such as incorrect illness perceptions, pain catastrophizing, anxiety and depression could contribute to and sustain the mechanisms of CS [70]. These associations may be bidirectional. Theoretically, neuroplastic changes originating from nociceptive pathways may spread to some brain areas like the insular, cingulate, prefrontal cortex, and limbic system, leading to pain catastrophizing thoughts, anxiety and depression [70]. These symptoms can increase forebrain activity, leading to the enhancement of central hyperexcitability and sensitization, which results in a vicious cycle leading to chronic pain and disability [19, 62, 70–72]. It is important to mention that many of the items on the CSI part A are common elements of anxiety and depressive disorders. Furthermore, we only used part A of the CSI in our study and our patients were maybe more likely to have reported depression or anxiety as a previous CSS diagnosis on CSI part B. It is worth noting that there is a significant overlap of symptoms between these conditions and central sensitization [73], and there is no set of established and scientifically recognized CS-defining criteria [74].

Our regression analysis showed that depression significantly predicted CSI scores in both KOA and CLBP groups. This result is further supported by the findings of Gervais et al. in KOA patients [64] and those of Miki et al. in CLBP patients [44]. Depression may enhance facilitatory pathways in the central nervous system, resulting in sensitization of dorsal horn spinal cord neurons [19, 75]. Anxiety and pain catastrophizing have also been described as one of the modulating factors associated with alterations in supraspinal endogenous pain inhibitory and facilitatory processes, thus maintaining and or aggravating CS pain [76, 77]. Although they were not found to directly predict CSI in our study, anxiety and pain catastrophizing might have an indirect influence on CSI through depression, to which they are highly associated.

Disease duration showed no significant association with CSI in both groups. This is consistent with previous findings in KOA and CLBP patients [43, 48]. Controversially, other studies reported a significant association between clinical pain duration and CS measurements in KOA [5, 78], and CLBP [13]. We might think that the KOA disease or the CLBP must be present for a sufficient period of time and/or have a sufficient degree of tissue injury for central sensitization to occur [13, 78]. Indeed, it has been postulated that persistent activation of peripheral nociceptors may ultimately lead over time to neuroplastic changes within the central nervous system [79, 80]. However, the lack of association of CSI with disease duration in our study, or with the Kellgren-Lawrence radiographic severity grade of KOA suggests that sensitization might be considered as a trait rather than a state, indicating that some individuals may be predisposed to central sensitization irrespective of the duration of their pathological condition.

In our study, the level of physical activity and sedentary behavior were not associated with the CSI in any group. Moriki et al. reported the same finding in a CLBP population [68]. However, previous studies had suggested that physical activity beneficially affected the functioning of the descending pain modulatory systems and facilitatory processes [81], and increased pressure pain tolerance [82]. Further research is needed to establish the relationship between central sensitization and physical activity.

A number of implications arise from the present study’s findings. First, the use of a simple method of sensitization assessment (the CSI) in clinical practice during the initial evaluation, the rehabilitation program, as well as the regular follow-ups may help identify KOA and CLBP patients at risk of a greater pain severity and who may potentially need pain medications targeting the central nervous system (e.g., anticonvulsants; selective serotonin and norepinephrine reuptake inhibitors [71, 83]). Second, psychosocial symptoms such as pain catastrophizing thoughts, anxiety and depression should also be considered as they are highly associated with CS in KOA and CLBP patients. Specific interventions including cognitive behavioral therapies [84] and neuroscience education [85] could be integrated into the treatment plan of these patients, which would more likely lead to better outcomes [86–89]. Further research would help in providing evidence of the utility of CSI as a treatment-outcome assessment tool after addressing the underlying factors involved in CS in KOA and CLBP patients, and explore whether improvements in CSI scores are associated with reduced pain and improved functioning in these patients.

Several limitations should be considered when interpreting this study’s findings. First, the design was cross-sectional; therefore, it is not possible to draw conclusions regarding causation or predictive validity. Second, the majority of surveys in this study relied on self-reported questionnaires, which raises the possibility of recall bias or overestimation of the indices being measured. Future studies will require more objective and quantitative methods for further verification. Third, the relationships between CSI and the somatosensory function measured by QST are still unknown in this population. In fact, the lack of consensus regarding the correlation between the CSI and QST in the existing literature suggests that the CSI may not reflect a direct measurement of CS [64]. Therefore, further longitudinal studies that include more objective pain sensitivity tests using a mechanism-based approach such as pain tolerance thresholds, spatial and temporal summation, conditioned pain modulation (CPM), spreading sensitization, and offset analgesia [14, 19, 90, 91] and examine their association to the CSI will be required to determine its accuracy in identifying pain processing changes involved in CS in KOA and CLBP patients.

## Conclusion

Our study found that central sensitization, as assessed by CSI, has a significant impact on pain, function and physical performance in KOA and CLBP patients. This outcome endorses the relevance of early CS assessment and management in KOA and CLBP patients in order to prevent transition into a chronic pain state. Moreover, the highly significant correlation of depression, anxiety and pain catastrophizing with CSI should be interpreted as a call for better understanding of these psychosocial factors affecting the patients’ pain experience to allow for a more focused and individualized treatment.

Further research would aid in providing evidence of the utility of CSI as a treatment-outcome assessment tool after addressing the underlying factors involved in CS in KOA and CLBP patients, and explore whether improvements in CSI scores are associated with reduced pain and improved functioning in these patients.

## Data Availability

The datasets used and/or analyzed during current study are available from the corresponding author on reasonable request.
